# Tipping the balance towards long-term retention in the HIV care cascade: A mixed methods study in southern Mozambique

**DOI:** 10.1371/journal.pone.0222028

**Published:** 2019-09-27

**Authors:** Laura Fuente-Soro, Carlos Iniesta, Elisa López-Varela, Mauro Cuna, Rui Guilaze, Maria Maixenchs, Edson Luis Bernardo, Orvalho Augusto, Raquel Gonzalez, Aleny Couto, Khatia Munguambe, Denise Naniche

**Affiliations:** 1 Centro de Investigação em Saúde de Manhiça (CISM), Maputo, Mozambique; 2 ISGlobal, Hospital Clínic—Universitat de Barcelona, Barcelona, Spain; 3 Centro Nacional de Epidemiología, Instituto de Salud Carlos III, Madrid, Spain; 4 Direcção Distrital em Saúde, Manhiça, Maputo, Mozambique; 5 National STI-HIV/AIDS Program, Ministry of Health, Maputo, Mozambique; 6 Faculty of Medicine, Eduardo Mondlane University, Maputo, Mozambique; University of the Witwatersrand, SOUTH AFRICA

## Abstract

**Background:**

The implementation of quality HIV control programs is crucial for the achievement of the UNAIDS 90-90-90 targets and to motivate people living with HIV (PLWHIV) to link and remain in HIV-care. The aim of this mixed method cross-sectional study was to estimate the linkage and long-term retention in care of PLWHIV and to identify factors potentially interfering along the HIV-care continuum in southern Mozambique.

**Methods:**

A home-based semi-structured interview was conducted in 2015 to explore barriers and facilitators to the HIV-care cascade among individuals that had been newly HIV-diagnosed in community testing campaigns in 2010 or 2012. Linkage and long-term retention were estimated retrospectively through client self-reports and clinical records. Cohen's Kappa coefficient was calculated to measure the agreement between participant self-reported and documented cascade outcomes.

**Results:**

Among the 112 interviewed participants, 24 (21.4%) did not disclose their HIV-positive serostatus to the interviewer. While 84 (75.0%) self-reported having enrolled in care, only 69 (61.6%) reported still being in-care 3–5 years after diagnosis of which 17.4% reported having disengaged and re-engaged. An important factor affecting optimal continuum in HIV-care was the impact of the fear-based authoritarian relationship between the health system and the patient that could act as both driver and barrier.

**Conclusion:**

Special attention should be given to quantify and understand repeated cycles of patient disengagement and re-engagement in HIV-care. Strategies to improve the relationship between the health system and patients are still needed in order to optimally engage PLWHIV for long-term periods.

## Introduction

Despite multiple efforts to scale-up universal antiretroviral therapy (ART) coverage, challenges remain in retaining people living with HIV (PLWHIV) in lifelong treatment and thus in achieving durable viral suppression and preventing transmission [1]. Attaining the UNAIDS 90-90-90 targets [2] depends on successful quality HIV National Health programs and on adherence of PLWHIV to ART [3].

In low-resource settings such as Mozambique, with an estimated 13.2% adult HIV prevalence in 2016 [4], indicators for linkage and retention in HIV-care remain lower than the targets. Analysis of National programmatic data in 2015 showed that 44% of individuals on ART were in-care after three years of follow-up [5]. The existing literature from sub-Saharan Africa has identified complex and multi-faceted barriers impeding optimal linkage and retention in the HIV-care continuum during the first year post-diagnosis. Barriers included stigma, health service access, drug side effects, abusive treatment by health providers or transport cost [6–12]. However, little is known about mechanisms through which specific individual, social and clinical level barriers affect long-term retention (i.e.>3 years) in care in the sub-Saharan Africa context, mainly due to challenges with paper based patient chart systems, poor census data and high mobility.

The aim of this mixed method study was (i) to estimate the linkage and long-term retention in care of individuals who newly tested positive during two cross-sectional community-based serosurveys performed in 2010 and 2012 and (ii) to identify the main factors interfering with each of the steps of the HIV-care continuum, in a semi-rural area of southern Mozambique.

## Materials and methods

### Study area and population

The study was conducted in the Manhiça district, Southern Mozambique, where the Manhiça Health Research Center (CISM) has continuously maintained, since 1996, a health and demographic surveillance system (HDSS) that captures vital events including migrations, pregnancies, births and deaths [13–15]. In 2015, during the study period, the HDSS covered a total population of approximately 174,000 inhabitants. The Manhiça district is a high burden HIV setting with an estimated community HIV prevalence among adults of 39.7% (95% CI 36.0–43.5) based on data from two cross-sectional community-based serosurveys conducted in 2010 and 2012 [16,17], which is unequally distributed across socioeconomic strata [18]. HIV services are offered free of charge and routine patient HIV-clinical data is recorded in an electronic Patient Tracking System (ePTS)[19]. Further clinical details in Supporting information (S1 Appendix).

### Study design and definitions

The current mixed method cross-sectional study included PLWHIV who had received a new HIV-diagnosis during either one of the two previous cross-sectional community-based serosurveys performed in 2010 and 2012 in the Manhiça District. These two serosurveys included a random sample of adults living in the HDSS aged 18 to 50. Details are described elsewhere [16,17].

Prior to the current study and in order to exclude non-eligible participants, a standard deterministic and probabilistic record-linkage [20], validated by other studies [21,22], was conducted to match individuals from the cross-sectional community-based serosurveys and the Manhiça District Hospital ePTS (MDH-ePTS) to identify individuals who were already known HIV-positive at the time of the 2010 and 2012 serosurveys.

Eligible individuals were visited at home from August-December 2015. After informed consent, a specifically designed questionnaire and a semi-structured interview was conducted. For further details on the questionnaire and the interview see the Supporting Information (S2 Appendix and S1 Fig).

For the purpose of the current analysis, the following definitions were used:

- **Linkage to care:** individuals who attended a first visit with a clinician for staging and/or evaluation of eligibility for ART.
- **Self-reported retention in care:** individuals that, at the time of the 2015 interview visit, self-reported being active in HIV-care. If they reported not being in-care, they were considered lost to follow-up (LTFU).
- **Documented retention in care:** defined as having a clinical visit registered in the MDH-ePTS in the 150 days or 180 days prior to the 2015 interview according to whether the participant had started or not ART respectively. This definition was constructed based on a proposed conservative universal definition of LTFU [23], which corresponds to being at least 90 days late for a clinical appointment.

### Quantitative data collection and analysis

Vital status for eligible individuals was extracted from the HDSS, and households of those deceased were not visited. During home-visits, sociodemographic information and clinical information regarding HIV follow-up were recorded for all interviewed participants in the electronic questionnaire in Open Data Kit software 1.4 (ODK)[24] and uploaded into a REDCap database (Research Electronic Data Capture) Software 5.7.3 [25]. To ascertain HIV status of the participants, interviewers first explored patient's knowledge on HIV and AIDS and then asked about previous HIV-testing history and result and in order to give the maximum opportunities to all participants to disclose their HIV status, at 3 stages of the interview were asked about their serostatus. For further details on the interview see S2 Appendix and S1 Fig in the Supporting Information.

The following steps of the HIV-care cascade were measured using two different data sources (self-reported care status and documented status at the MDH-ePTS): 1) enrollment in care, 2) first clinical consultation, 3) clinical or laboratorial WHO HIV-staging, 4) ART criteria and 5) retention in care. The self-reported cascade of care was constructed within the time frame between diagnosis and the household visit while the documented cascade of care was constructed between diagnosis and a 36-months post-diagnosis.

Descriptive analyses of sociodemographic characteristics stratified by year of diagnosis, assessing difference in proportions by Pearson and Fisher’s exact chi-square tests were conducted. Wealth index was constructed through a combination of HDSS variables as previously described [18].

Cohen's Kappa coefficient was calculated for each step of the cascade to measure the percentage of agreement between participant self-reported and ePTS documented cascade outcomes [26]. Only individuals that self-reported being registered in the MDH were included. Self-reported and documented cascades were administratively censored at the time of the current interview visit.

### Qualitative data collection and analysis

Interviews were conducted to all participants in Changana (local language) or Portuguese, according to participant preference, and lasted approximately one hour. Three local experienced HIV-counselors trained by the local social science team performed the interviews, digitally audio-recorded, transcribed, translated and summarized them into Portuguese.

A purposive sub-sample of interviewed participants was selected according to criteria described in the literature to be associated with retention and loss to follow up in care in order maximize the variety of the expressed barriers/facilitators These factors included gender, age, educational level, year of diagnosis, distance to the health facility and HIV-care status (in-care/out-of-care)[27–31]. From the possible theoretical combinations of these variables, individuals who fulfilled those profiles were identified, and up to two participants were selected for each profile in order to reach a representative sample of the participants for the qualitative analyses.

Following methodology for a framework approach based on both inductive and deductive thematic analysis [32], an initial codebook, based on literature on barriers to linkage and retention in care [11,27–31, 33–42], was applied while reading and simultaneously coding the first transcripts and new codes were added as they were emerging from the interviews. Coding entailed identifying passages of text related to the topics of interest and labeling them with those specific codes. Three HIV researchers independently performed manual coding and tabulated all passages of text related to the pre-determined and emerging codes from selected interviews into a matrix format using MS Excel.

According to conceptual similarities, codes were grouped into subcategories and categories, and related categories into main levels of influence. Discussions were built around each level and around links between them, and from these discussions, the main conclusions emerged. To ensure data quality, discrepancies in codification and categorization were discussed several times by the three researchers and reclassification of codes, transcripts and categories were done if necessary.

### Ethics

This study was approved by the Mozambican National Bioethics Committee as well as the Institutional Review Boards at the Hospital Clinic of Barcelona (Spain) and the Manhiça Health Research Centre. The purpose of the study was explained to participants and written informed consent was obtained.

## Results

### Study profile and baseline characteristics

**Fig 1:**
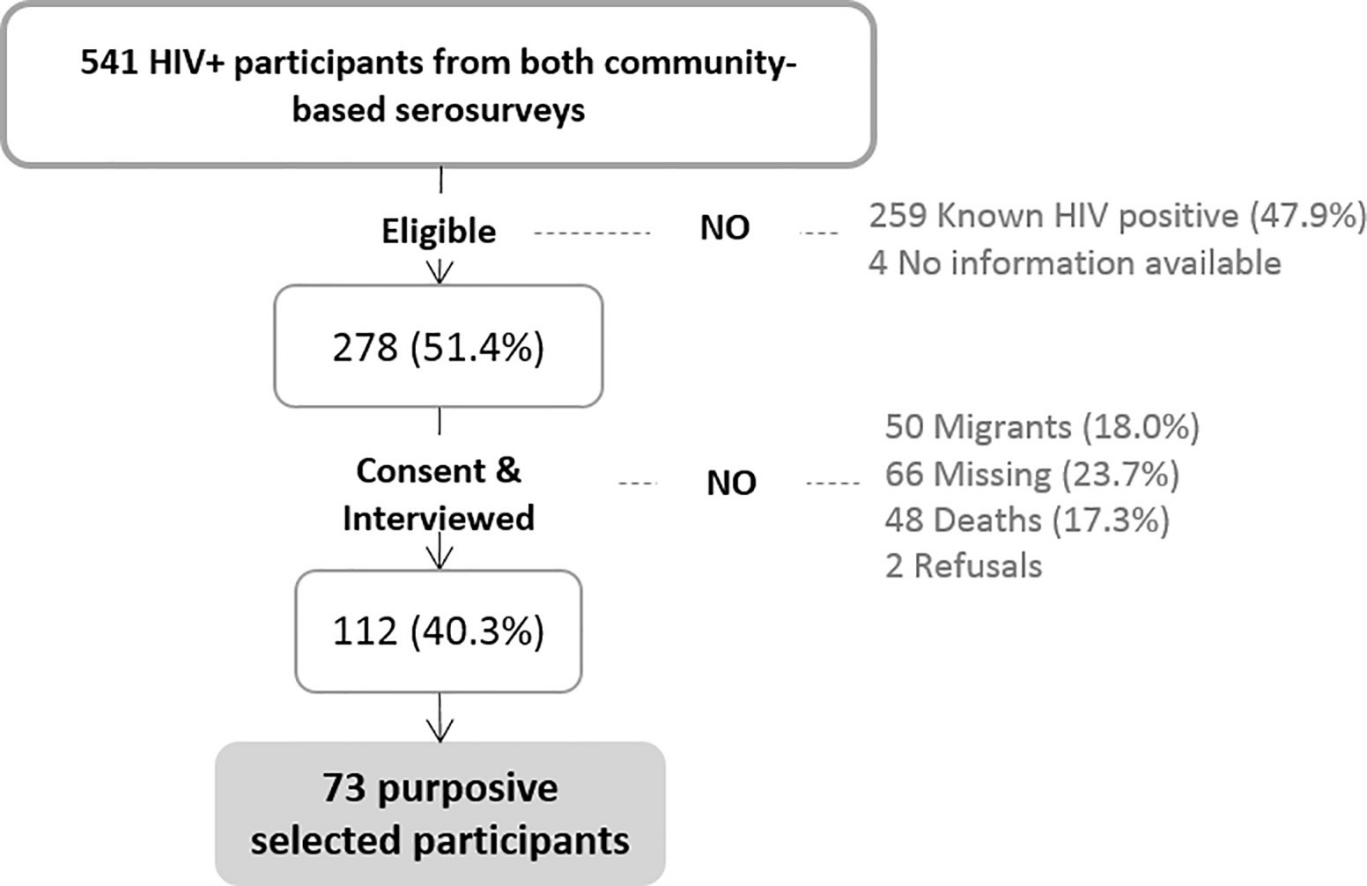
Study profile for participants who tested HIV positive during the cross-sectional community-based serosurveys in 2010 and 2012. Percentages are calculated over the previous step.

From the total of 541 individuals who tested positive in either cross-sectional community-based serosurvey (Fig 1), 259 (47.9%) were identified as known HIV-positive because they were registered in the MDH prior to the 2010 and 2012 serosurveys and thus, excluded. From the remaining 278 (51.4%) individuals considered newly diagnosed, 48 (17.3%) were deceased at the time of the current study, 66 (23.7%) were absent from the household and 50 (18.0%) had migrated out of the study area.

Mortality rate between diagnosis and study procedures among the 278 newly diagnosed individuals was 4.2 per 100 person-years and median time from diagnosis to death was 23.5 months (IQR 9.3–33.5), with no significant differences by year of diagnosis (p = 0.7333).

A total of 112 (40.3%) were visited and willing to participate and to be interviewed (Fig 1). Among them, 24 (21.4%) did not recognize their HIV-positive serostatus to the interviewer and refused to be re-tested for HIV. Globally, 52.7% had been diagnosed in 2010 and 47.3% in 2012 (S1 Table). Baseline socioeconomic characteristics did not differ by year of diagnosis except for level of education (p = 0.020).

### Self-reported cascade of care

**Fig 2:**
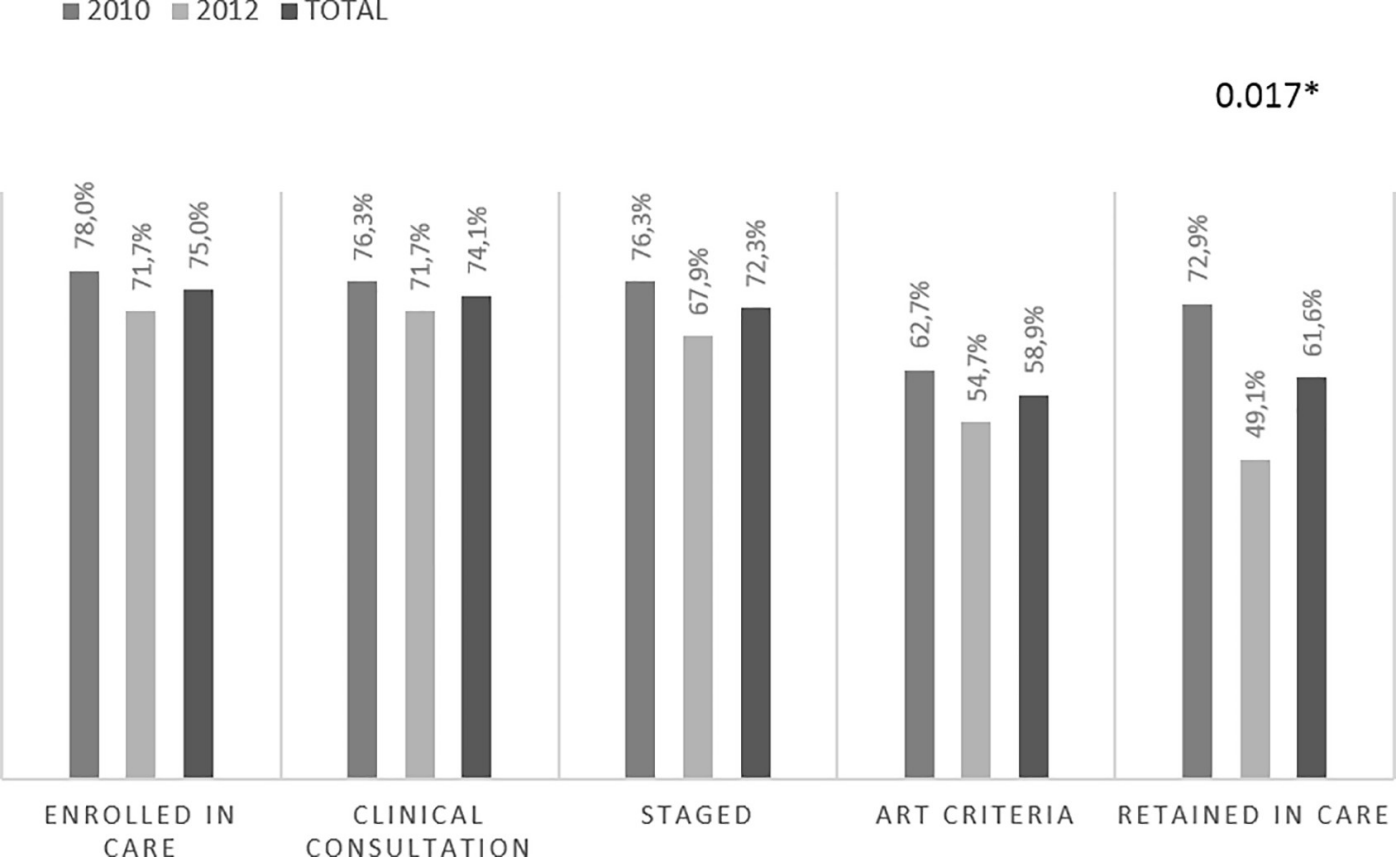
Self-reported cascade of care among interviewed participants according to year of diagnosis. Self-reported cascade was constructed based on participant responses to the interview and within the time frame between diagnosis and the time of the interview visit. Thus, individuals diagnosed in 2010 reported around 5 years of follow-up while individuals from 2012 reported around 3 years. Each bar represents the proportion of the total number of individuals interviewed (n = 112) by step of the cascade. Abbreviation: Anti-retroviral therapy (ART).

Eighty-four (75.0%) participants reported being enrolled in care in any health facility while the other 25% reported never enrolled since their HIV-diagnosis. Of those enrolled, 68 individuals (80.9%) denoted the MDH as their referral health center, while 16 (19.1%) were registered in other province units. As shown in Fig 2, 74.1% participants reported at least one clinical consultation after diagnosis and 72.3% reported at least one visit to the laboratory unit for staging. Only 66 (58.9%) of the individuals visited by a clinician had started ART treatment and 69 (61.6%) reported being in-care at the time of the study interview (Fig 2).

Individuals diagnosed in 2012 had a shorter FU time and lower proportion were in-care as compared to those diagnosed in 2010 (49.1% vs 72.9%, p = 0.017 respectively, Fig 2). Among the 16 participants who self-reported being LTFU at the time of the interview, a significantly higher proportion were ART-ineligible as compared to ART-eligible participants (56.2% vs 43.7% respectively (p<0.001)).

Finally, twelve of the 69 individuals in-care at the time of the interview (17.4%) reported having disengaged and re-engaged at some point of their follow-up, without differences by year of diagnosis.

### Cascade of care: Self-reported versus documented

**Fig 3:**
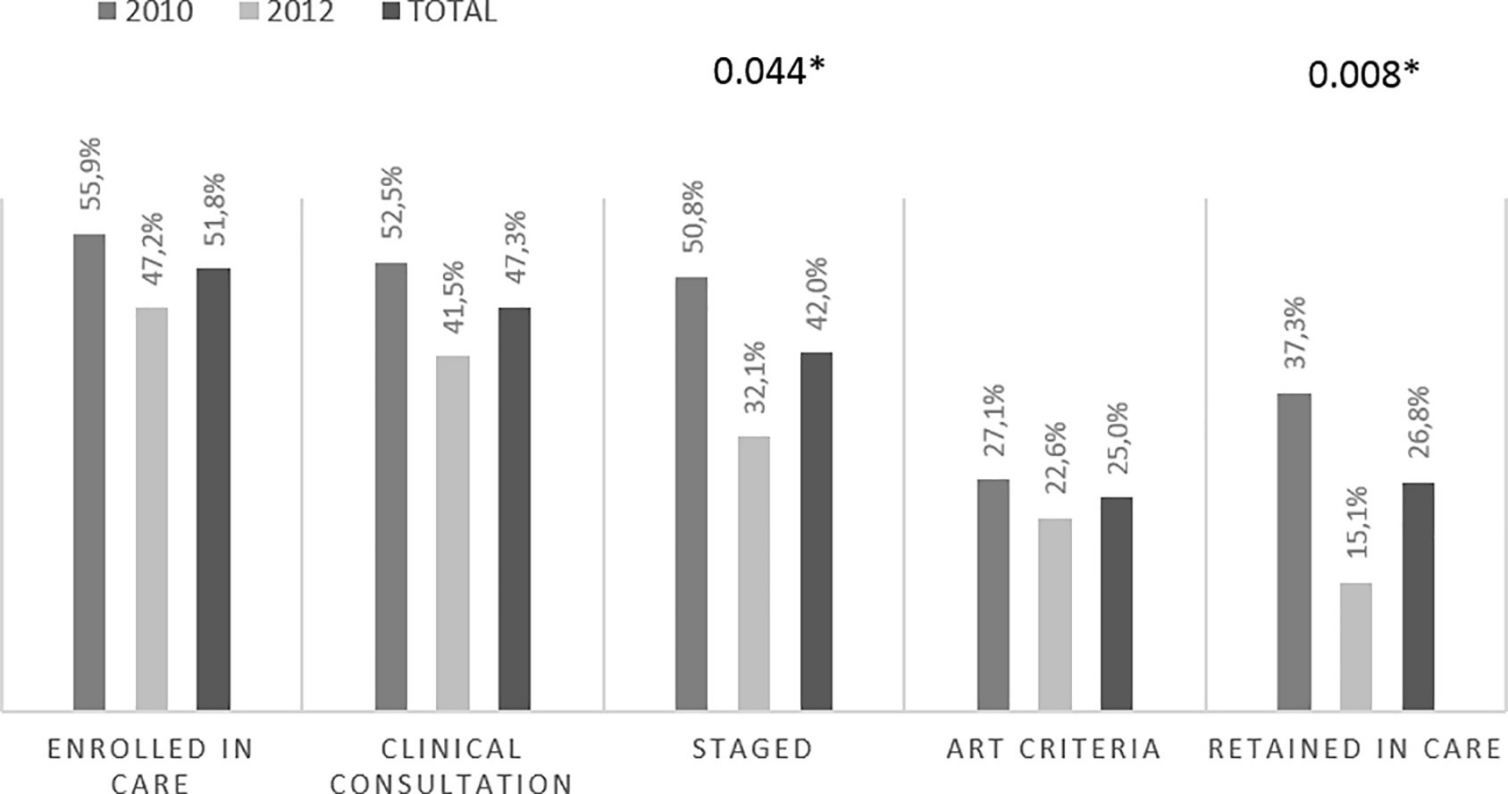
Documented cascade of care after 36 months post-diagnosis among interviewed participants (n = 112) according to year of diagnosis. Documented cascade was constructed based on clinical information extracted from the MDH-ePTS. All participants were administratively censored at 36-months post-diagnosis. Each bar represents the proportion of the total number of individuals interviewed (n = 112) by step of the cascade. Abbreviation: Anti-retroviral therapy (ART).

Regarding the documented cascade, 65 out of 112 participants were registered in care in the MDH-ePTS after their HIV-diagnosis (Fig 3) of which, only 58 (89.2%) were enrolled within 36 months post-diagnosis and only 28 (48.3%) of those had started ART. Overall, individuals diagnosed in 2010 were more likely than those diagnosed in 2012 to have been staged (50.8% vs 32.1%, p = 0.044) and to be retained in care (37.3% vs 15.1%, p = 0.008) after 36 months (Fig 3).

We then assessed the concordance between the self-reported and documented cascades for the MDH, thus, out of the total 112 individuals interviewed, only the 68 participants that self-reported being registered in the MDH were included. Kappa coefficients ranged between 0.62 to 0.76 between both cascades in all steps of the care continuum (S2 Table) showing a substantial agreement.

A total of 45.6% (36/79) of the individuals that were documented as LTFU by the MDH-ePTS database self-reported being in-care. However, none of the individuals documented to be retained by the MDH-ePTS self-reported being lost to follow-up.

### Factors influencing clinical HIV-care

Seventy-three participants were selected for qualitative analysis. Table 1 presents factors that emerged and that influenced the respondents’ navigation through all steps of HIV-care, from testing to linkage, retention and re-engagement after disengagement, as well as cross-cutting themes. Factors were coded and aggregated in 16 categories and grouped into four principal levels of influence: individual level, social level, related to the health system and related to the antiretroviral medication (Table 1 and S3 Table). Quotes are summarized in Table 2. The saturation of information in the categories did not warrant expanding the analysis to the additional participants interviewed.

**Table 1:** Distribution of main emerging factors along the HIV continuum of care.

	INDIVIDUAL LEVEL	SOCIAL LEVEL	RELATED WITH THE HEALTH SYSTEM	RELATED WITH ARV MEDICATION
	Health Literacy	Social support	Health System Compliance	Side effects of the ARTs
ALL	Competitive needs	Familiar/partner support	Health System approaches to increase linkage and retention to care	Wiliness to take ART
	Value of health status	Traditional healers/medicine	HIV-care workflow	ART stock outs
	Other limiting situations	Empowerment of people living with HIV	Self-perception of disease control through CD4 counts	ART therapeutic options
				
	Health Literacy	Social support	Health System Compliance	
FROM TESTING TO LINKAGE	Competitive needs	Familiar/partner support	Health System approaches to increase linkage and retention to care	Wiliness to take ART
	Value of health status	Traditional healers/medicine	HIV-care workflow	
	Other limiting situations	Empowerment of people living with HIV		
	Health Literacy	Social support	Health System Compliance	Side effects of the ARTs
LONG-TERM RETENTION	Competitive needs	Familiar/partner support	Health System approaches to increase linkage and retention to care	Wiliness to take ART
	Value of health status	Traditional healers/medicine	HIV-care workflow	ART stock outs
	Other limiting situations	Empowerment of people living with HIV	Self-perception of disease control through CD4 counts	ART therapeutic options
		Social support	Health System Compliance	
RE-ENGAGMENT AFTER BEING LOST TO FOLLOW-UP		Familiar/partner support	Health System approaches to increase linkage and retention to care	Wiliness to take ART
	Value of health status		HIV-care workflow	
	Other limiting situations			

**Table 2:** Selected levels of influence and quotes from study participants. Factors that influence the uptake and the long-term engagement in HIV care are represented.

	SUB-CATEGORY/CATEGORY	BARRIER OR FACILITATOR	
**FROM TESTING TO LINKAGE**		** **	
**1. INDIVIDUAL LEVEL**	Empowerment/Reduction of HIV-related stigma	*"I didn't go because I was sick*, *I went because I saw that because this disease is too frequent*, *let me make the test* .* *.* *. *if not*, *(if I don't do it) I will stay (without doing anything) while I have it (while I'm HIV positive)" "I realized that HIV is the disease of the century*, *maybe I can have it*. *So I went (to do test)*!*" [Diag 2010*, *F*, *49]*	
**2. SOCIAL LEVEL**	Support: Partner (lack of)	*"I started the process (enrol in care)*, *but I didn't take the medication* .* *.* *. *because they told me that I had to tell my husband to test him too*, *but he refused that I continue with the treatment"*, *[Diag 2012*, *F*, *26]*	
**3. INDIVIDUAL LEVEL**	Illness/Other limiting situations	*"I started getting sick*, *I fell down and I started to suffer from pain in my limbs*, *my mouth turned*, *my children accompanied me to Maragra's hospital" [Diag 2010*, *M 50]*	
**4. RELATED WITH THE HEALTH SYSTEM**	Hospital: Need to return to hospital another day	*"The following happend*: *the day they told me I had to go*, *I was not* .* *.* *. *(*.* *.* *.*) I had found a place to work that I could not even leave*, *I did not have time (*.* *.* *.*)*, *so on the day that I was scheduled to go to the hospital*, *I failed*. *I went the other day and they said "back" to go start again*, *so I couldn't I never come back again"*, *[Diag 2012*, *M*, *40]*	
**5. RELATED WITH THE HEALTH SYSTEM**	Fear of being scolded in hospital/Health System Compliance	*"When I arrived to the hospital*, *I left the guide (testing documentation) with the receptionist*. *I left it in the window*, *and they received it and went to do other work at the reception*. *I didn't ask anything because they scared us in the hospital*, *how can I ask if we were so many*?*"*, *[Diag 2012*, *M*, *43]*	
**LONG-TERM RETENTION**		
**6. INDIVIDUAL LEVEL**	Health perception/Health Literacy	*"I want to continue with the follow up because I'm seeing the results" "Before*, *I always felt my body weakened*, *but now I feel good"*, *[Diag 2010*, *M*, *53]*	
**7. INDIVIDUAL LEVEL**	Value of own health	*"Other brothers die because they do not take care of their health*, *they like to abandon because a lot of things get spoiled*, *they start saying aaaaaah*, *hospital does not work*, *they do not treat us at the hospital* .* *.* *.*" [Diag 2012*, *M*, *53]*	
**8. INDIVIDUAL LEVEL**	Parenthood/Health Literacy	*"Follow-up at the hospital is important because I have small children* .* *.* *. *when I die*, *no one will be caring for my daughters* .* *.* *. *I have to continue with the treatment in order to raise my children"*, *[Diag 2010*, *F*, *25]*	
**9. RELATED WITH THE HEALTH SYSTEM**	Fear of being scolded in hospital/Health System Compliance	*"* .* *.* *. *I went (to the hospital)*, *but then I traveled and then they stole my bag and I always walked with my hospital card* .* *.* *. *so they stolen me mycard* .* *.* *.*" "I didn't went anymore* .* *.* *. *I'm afraid because I don't have my card (hospital identification)* .* *.* *. *Someone told me that when you miss the appointments*, *they talk* .* *.* *. *"*, *[Diag 2012*, *F*, *22]*	
**10. RELATED WITH ARV MEDICATION**	Side effects of the ARTs	*"I was in care for a while but one day was time for me to start with the pills and then I gave up*, *I did not take it because I did not feel any pain in my body*, *and I heard that when you take the medicines while you are healthy you can get sick" [Diag 2012*, *M*, *44]*	

#### 1. From testing to linkage to care

When asked about facilitators at the first steps of the cascade, several respondents, especially women, reported the value of the health status, expressed as the desire to live and to be healthy. Moreover, they also expressed fear of the disease and many participants shared stories about HIV-positive relatives/friends that had delayed seeking care and had become sick or died. Other key facilitators prompting the seeking of care, beyond the value of the health status, were reported by several participants that went to the health facility in order to know their HIV serostatus and start care if necessary after realizing that “HIV is the disease of the century” and that it “affects us all” (Quote 1 [Diag 2010, F, 49]). Important differences by gender were found in terms of empowerment and social or family support. Women faced the diagnosis and continued follow-up on their own, or with the support of other women, and encouraged their partners to seek diagnosis. Despite the observed female support, several women reported their partner’s refusal to be followed up as a barrier (Quote 2 [Diag 2012, F, 26]). On the other hand, many men described being motivated by their wives or other female relatives to seek care.

Competing needs such as migration/mobility, seasonal work in South Africa or having to travel to attend funeral ceremonies of a relative, were reported by some participants as a barrier for not linking to care after diagnosis. Delayed access to HIV-diagnosis and treatment was common and severe illness seemed to be a motivator to initiate care at the time of diagnosis (Quote 3 [Diag 2010, M, 50]). Barriers related with the health system were also reported. Although many participants felt confident with clinicians and stated that the hospital is the only place where one’s health can be improved, they faced many structural challenges that made them drop out (long queues and waiting times, incompatibility with working hours, feeling of being lost during the hospital circuit, etc). After diagnosis, patients said they had multiple visits to the health facility in order to be seen by the clinician (Quote4 [Diag 2012, M, 40]). Moreover, they expressed frustration and fear because of the lack of empathy and disrespectful behavior of health care providers throughout their initial visits (Quote 5 [Diag 2012, M, 43]).

#### 2. Long-term retention in care

Participants described their perceived health improvement as one of the most relevant individual factors that motivated them to continue HIV-care. Many, especially those on ART, had experienced negative health outcomes in the past followed by improvements after initiating care. They thus valued adherence to care, seeing it as essential to avoid returning to their previous state (Quote 6 [Diag 2010, M, 53]). Moreover, several respondents were familiar with the possible adverse health outcomes associated with inadequate HIV-care (Quote 7 [Diag 2012, M, 53]). The desire to live was also reported as an important driver of remaining in care. Additionally, the desire to be alive to take care of their offspring was expressed exclusively by women (Quote 8 [Diag 2010, F, 25]).

One of the most significant factors affecting the continuation in care, in a bivalent way, was the strong authoritarian relationship observed between health providers and the patient. On one hand, the vertical relationship between health provider and patient seemed to be a driver of compliance with the HIV care recommendations, motivate long-term retention, and pressure clients to comply with “the hospital laws” in order to be healthy. Several participants explained that their motivation to continue in care was the confidence in the clinician and duty to abide by rules of the health system. *"It is my duty (to continue in care)*, *I have to comply*, *do what the doctor tells me to do" (Diag 2012*, *F*, *44)* explained a woman. On the other hand, this same feeling of need to comply with the hospital laws also hindered continuation in care as several patients reported dropping out for fear of coming back to the health facility after failing to attend one appointment or losing their hospital identification card (Quote 9 [Diag 2012, F, 22]). Moreover, a women said that *"I didn't come back to the hospital because*, *after going to South Africa*, *I was afraid to return*. *I fail my last appointments* … *I couldn't come back*, *I‘m afraid*” (Diag 2010, F, 30).

Secondary effects of ART medication emerged as a barrier that had been overcome. Many interviewees expressed having suffered symptoms after starting ART, however they emphasized the improvements in their quality of life after a certain period of time and none of them had dropped-out because of the negative drug side effects. Only one participant (Quote 10 [Diag 2012, M, 44]) refused treatment initiation because of the misconception that if the medication is taken while you “are healthy”, it will make you ill.

## Discussion

Results of this mixed-method study, conducted in southern Mozambique, among newly HIV-diagnosed individuals contribute to understand the main factors that influence PLWHIV’s linkage and retention in care. During the home-based interviews, 40.3% of the eligible participants were traced and interviewed. Among those, 24% of HIV-positive individuals, did not disclose their positive serostatus to the counsellor similar to what we previously described in the same area [21]. Furthermore, the self-reported and documented HIV-care cascade revealed that almost twofold more individuals were in-care among 2010 diagnosis as compared to 2012 diagnoses. A substantial correlation (kappa coefficient) was observed between the self-reported and health-system documented (ePTS) cascades of HIV-care. Additionally, after examining the multi-level factors that affected optimal linkage and retention in HIV-care, the impact of an authoritarian health system as both a driver and barrier emerged as a very important issues faced by individuals.

In individuals interviewed 3 years after diagnosis, 49.1% reported being in-care, similar to national estimates [5]. However, in individuals interviewed 5 years after diagnosis 72.9% reported being in care. This is counter-intuitive to the logic that the greater the time since diagnosis, the greater the attrition and points to a more complex relationship between HIV acquisition, diagnosis and long term-retention. A study conducted in France between 2004 to 2014 showed that the average delay between acquisition and diagnosis was 3 years [43]. In this study, individuals diagnosed 5 years earlier, many who had not sought care, were more likely to reach advanced disease, which has been shown to increase linkage and retention. On the other hand, a higher proportion of individuals in-care at 5 years, may reflect more time for attrition but also for re-engagement. Indeed, in our study, close to 20% of participants in-care reported having LTFU and re-engaged. Retention and LTFU are not mutually exclusive and attention should be given to repeated cycles of LTFU and re engagement.

Since the beginning of the HIV epidemic, the role of the health system as well as HIV stigma in all its dimensions have been considered as the most potentially influential barriers that PLWHIV face [44]. Stigma has been defined as i) anticipated, ii) enacted and iii) internalized or “self-stigma”. Fear of disclosure of HIV status has been described to be related with discrimination and rejection [45] and is considered a major consequence of anticipated stigma [8]. Similar to previous results in the region [21], our findings documented an elevated proportion of HIV positive individuals who did not disclose or who even denied their previous HIV status to a lay counsellor in a home-based interview. Although stigma may play an important role in non-disclosure, other reasons could include distrust in the result, desire to keep their health status private or fear of disclosing LTFU from care among other possible reasons. Interventions to reduce stigma and “normalize” HIV infection, have likely decreased enacted stigma and “active” discrimination but other forms of stigma may be more difficult to eradicate. Although our results did not reveal evidence of direct discrimination or enacted stigma, our results suggest that measuring the proportion of known HIV-positive individuals that do not disclose their serostatus to a counsellor during a home-based counseling session could be used as proxy to measure the progress in reducing anticipated stigma.

In our study, one of the important patient-reported cross-cutting issues that emerged through all steps of the cascade was the relationship between the health system and the patient, which had a bivalent and cumulative effect along the continuum. On one hand, responses from PLWHIV from our study reflected a strong authoritarian and vertical relationship where the health system was in the position of power and the patient occupied the lowest level and complied with the norms and laws [46] of the hospital. Indeed, studies conducted in Uganda and Sudan shown that less half of the patients and health providers are aware of the existence of national Charter of patients’ rights, increasing client vulnerability [47,48]. On the other hand, our results highlighted that these PLWHIV also expressed confidence in the health system. PLWHIV believed that they needed to be followed in the “formal” health system and they trusted clinicians to “cure” them. This reflects a complex mixture of confidence in the health system combined with fear generated by the rules-based power structure of health provision in many areas of sub Saharan Africa. In an authoritarian system where adherent PLWHIV are treated better than those who are not, fear of punishment can act as a driver for continuation in care for in some. However, patient non-compliance with rules for whatever reason, may lead to disrespect by the health worker and disengagement of the PLWHIV. A recent qualitative systematic review highlights the duality of an authoritarian health system construct as useful for continuation in care in some PLWHIV but mostly detrimental [49]. A factor which may whittle patient confidence in the health system is the perception of including insensitive healthcare worker attitudes and facility workflow constraints, that appeared in our results as influent barriers, similar to previous results from other studies [50,51]. Zanolini has described how PLWHIV in Zambia preferred waiting more than 19 hours or travelling 45 km in order to be attended by friendly health care providers rather than rude providers [52]. Differentiated care approaches have been implemented in order to overcome some facility workflow constraints with a positive impact on linkage and short/medium-term retention [52–54]. However, strategies that improve client-provider relationship must be developed to overcome this important barrier.

Our results found PLWHIV empowered, especially women, with a strong desire to live, acting as mediators in their communities and helping others to engage and remain in-care at the health facility, where the clinician could treat and keep them alive. As Camlin et al illustrated [55], a new therapeutic citizenship project is emerging and could influence in stigma reduction and in successful HIV-care cascade implementation. In this sense, long-term retention could potentially be improved if health facilities become a friendly place where PLWHIV feel comfortable and respected. Despite the empowerment reflected in the women’s responses, lack of support from their male partners emerged as an important barrier to care. Indeed, in a study conducted in Uganda, women who did not benefit from their spouse's support were nearly four times more likely to drop out of care than those who had supportive husbands [56]. In Mozambique, the need for a husband’s approval to start treatment poses a barrier for many women [57].

Finally, as we move towards HIV control and elimination, we will require accurate monitoring of evolving barriers to retention and of steps to overcome them. In countries with limited resources such as Mozambique, routine data is paper-based, and subsequently digitalized in the electronic database, as described elsewhere [58] and only gives quantitative clinical indicators of care. Our results suggest that cross-sectional studies like ours could be performed as an inexpensive approach to monitor the evolution of barriers, as well as to address retention from the self-reported community perspective as complementary to the documented facility-based approach.

Our study had some limitations. First, we did not interview health providers, administrators or other stakeholders who may have had additional insights and perspectives on barriers and facilitators of long-term retention of clients in HIV-care. Secondly, our study was not designed to specifically analyze stigma. Thus a deeper understanding of the causes of non-disclosure to health providers and their relationships with anticipated stigma is needed. Thirdly, due to the methodology used to identify newly HIV diagnosed individuals, some of the participants that at the time of the cross-sectional serosurveys knew their HIV-status but had not enrolled in care or were enrolled in other health facilities could have been misclassified as newly diagnosed. Furthermore, we cannot exclude that deceased participants from the previous surveys might have expressed different barriers to care. Moreover, although our results do not seem to point clearly to stigma as a barrier of long-term retention, we cannot dismiss its possible impact and further research to explore this specific association would be needed. Also, in investigating long-term retention and self-perceived engagement in care, we cannot exclude recall bias and potential social reconstruction of events combined with social desirability which could modify respondents’ perceptions about barriers or facilitators.

## Conclusion

Our results highlight the importance of the relationship between PLWHIV and the health system in the achievement of the 90-90-90 targets. Depending on the interaction, the health system can act as a double edged sword representing either a facilitator or a barrier across the cascade of care. The high proportion of PLWHIV who do not disclose their HIV status to the counsellor is likely to reflect anticipated stigma. Making patients feel their needs are heard, respected and addressed and that they are active participants in their HIV-care, and not simply passive users of the health system could be a possible approach in order to support long term retention. Cycles of LTFU and re-engagement will be important to measure as we assess retention in HIV-care for longer periods. And finally, cross-sectional studies like ours could be performed as an inexpensive approach to monitor the evolution of barriers in long-term retention in care, as well as address retention from the self-reported community perspective as complementary to the documented facility-based approach.

## Supporting information

S1 Appendix National guidelines on HIV clinical follow up. (DOCX)

S2 Appendix Study questionnaire. (DOCX)

S1 Fig Flowchart of the semi-structured interview performed for all eligible participants found and who consented (n = 112). (DOCX)

S1 Table Baseline sociodemographic characteristics of the study population according to year of diagnosis (n = 112). (XLSX)

S2 Table Comparison between the proportion of individuals interviewed who self-reported or where identified through the MDH-ePTS by step of the cascade. (XLSX)

S3 Table List of all emerging codes and how they are grouped in sub-categories, categories and levels of influence. (XLSX)
