# Supplementary material for: Tipping the balance towards long-term retention in the HIV care cascade: A mixed methods study in southern Mozambique
Source: PLoS One. 2019 Sep 27;14(9):e0222028. doi: 10.1371/journal.pone.0222028 (PMC6764678; doi:10.1371/journal.pone.0222028)
Supplement: S1 Appendix — (DOCX) [file pone.0222028.s001.docx]

**Supporting information**

**Methods**

**S1 Appendix. National guidelines on HIV clinical follow up**

National HIV guidelines at the time of both serosurveys, stipulated that HIV counseling and testing was performed mainly in the voluntary counseling and testing unit. After a positive HIV result, patients gone to the health facility reception in order to be enrolled in care and scheduled their first clinical visit. In the first consultation, the clinician scheduled CD4 testing which was performed at the MDH lab, normally after a few days due to restricted opening hours. The patient was responsible for collecting the results prior to the next clinical consultation. Once HIV positive individuals enrolled in care, it was recommended that they returned regularly to the health facility in order to receive adherence counseling, clinical and laboratorial assessment and medication if needed. At the time of HIV testing, national criteria to start ART was CD4 count less than 250 cell/mm^3^ for patients in WHO stage I and II, 350 cell/mm^3^ for WHO stage III and all patients in WHO stage IV. Clinical appointments and laboratorial staging took place every two or three months during the first year post-diagnosis depending if the patient had criteria to start treatment or not, and every six months after the first year of follow up. ART pick-up was scheduled monthly (21).
