# Supplementary material for: Tipping the balance towards long-term retention in the HIV care cascade: A mixed methods study in southern Mozambique
Source: PLoS One. 2019 Sep 27;14(9):e0222028. doi: 10.1371/journal.pone.0222028 (PMC6764678; doi:10.1371/journal.pone.0222028)
Supplement: S2 Appendix — (DOCX) [file pone.0222028.s002.docx]

**Supporting information**

**Methods**

**S2 Appendix. Study questionnaire**

|  | **Participant information** | | | |  |
| --- | --- | --- | --- | --- | --- |
|  | **Perm_id** \|__\|__\|__\|__\| - \|__\|__\|__\| - \|__\|__\| | | | |  |
|  | **Check** digit \|__\| | | | |  |
|  | **CARE Study number - \|__\|__\|__\|__\|** | | | |  |
|  | **Did the participant have a Hospital ID?** □ Yes □ No  4.1 If Yes: number \|__\|__\|/\|__\|__\|__\|/\|__\|__\|/ \|__\|__\|__\|__\|__\| | | | |  |
|  | **House located** □ Yes □ No  5.1 If Yes, household number \|__\|__\|__\|__\|-\|__\|__\|__\|  5.2 If No, why? □ House not found □ Destroyed house  □ Abandone house □Other \|__\|__\|__\|__\|__\|__\|__\| | | | |  |
|  | **Participant located** □ Yes □ No  6.1 If No, why? □ Absence □ Death □ Migration □ Other \|__\|__\|__\|__\|__\|__\|__\|__\|__\|__\|__\| | | | |  |
|  | **Did the participant sign the informed consent**  □ Yes □ No  7.1 If No, why? □ Refusal □ Partner refusal □ Other \|__\|__\|__\|__\|__\|__\|__\|__\|__\|__\|__\|  ***If the participant refused the participation in the study, end the questionnaire here*** | | | |  |
|  | Sociodemographic information | | |  |  |
|  | **Level of education:**   - unschooled - Literacy - Primary school - Secondary school | - University - Don’t know/Don’t answer - Other \|__\|__\|__\|__\|__\|__\|__\|__\|__\|__\|__\| | | |  |
|  | **Primary occupation:**   - Administration - Agricultural - Farmer in his own orchard. He does not trade - Carpenter - Charcoal burner - Clinician - Merchant (with fixed salary) - Domestic (own house) - Electrician - Housekeeper (in another house) - Student - Guard/security - Disabled - Lumberjack - Military/Police | | - Miner in South Africa - Fixed vendor (market) - Peddler - Servant - Teacher - Driver - Doesn’t work - Bricklayer - Fisherman - Retired/Pensioner - Religious - Locksmith - Technician - Working in South Africa - Other \|__\|__\|__\|__\|__\|__\|__\|__\|__\|__\|__\| | |  |
|  | **Type of religion** \|__\|__\|__\|__\|__\|__\|__\|__\|__\|__\|__\|__\|__\|__\|__\|__\|__\|__\| | | | |  |
|  | **Does the participant currently have a partner?** □ Yes □ No  11.1 If Yes, how long ago? \|__\|__\| □ Months □ Years □ Days  11.2 Do you live with him/her in this house? □ Yes □ No | | | |  |
|  | **Does the participant have children?** □ Yes □No  12.1 If Yes, how many? \|__\|__\|  12.2 If Yes, how many < 5 years \|__\|__\| | | | |  |
|  | Knowledge on HIV | | | |  |
|  | **Have you ever heard of HIV and AIDS?** □ Yes □ No | | | |  |
|  | **Can you tell me what you know about HIV and AIDS?**   - *Explore the following aspects:*   - *Transmission*   - *Prevention*   - *If there is a cure*   - *What causes AIDS* | | | |  |
|  | Diagnosis information | | | |  |
|  | **Has the participant ever been tested for HIV?** □ Yes □ No □ Don’t know | | | |  |
|  | If 16 is Yes, what was the HIV test result? □ Positive □ Negative □ Don’t know | | | |  |
|  | 16.1 Where does the participant report having been tested for HIV?  □ Home □ VCT □ Primary care/PICT □ Maternity □ Urgency □ Don’t know  □ Other, specify \|__\|__\|__\|__\|__\|__\|__\|__\|__\|__\|__\| | | | |  |
|  | ***If the participant says he/she do not know his/her HIV status or says that it is negative, try to remember when he/she were tested and ultimately, after counseling, offer the HIV test***  □ Refuses to test □ Accepts the test  □ Acknowledges having been tested previously  17.1 If the participant acknowledges having been tested before, what was the result?  □ Positive □ Negative □ Don’t know  17.2 Where does the participant report having been tested for HIV?  □ Home □ VCT □ Primary care/PICT □ Maternity □ Urgency □ Don’t know  □ Other, specify \|__\|__\|__\|__\|__\|__\|__\|__\|__\|__\|__\| | | | |  |
|  | Access and linkage to HIV care | | |  |  |
|  | **Did the participant already open an HIV medical chart at any health facility (HF)? Did he/she enrolled in care?**  □ Yes □No □ Don’t know  18.1. If Yes, where? □ HF Manhiça □ HF Maragra □ HF Palmeira/Nwamatibjana  □ HF Xinavane □ HF Taninga □ Xai-Xai Hospital  □ HF Munguini □ HF Calanga □ HF 3 Fevereiro  □ HF Ilha Josina □ HF Xibukutsu □ HF Malavel  □ HF Maluana □ HF Marracuene □ Maputo City  □ Other \|__\|__\|__\|__\|__\|__\|__\|__\|__\|__\|__\| | | | |  |
|  | If 18 is Yes, what did he/she encouraged to enroll in care?   - *Explore the potential facilitators that helped the participant to enroll in care.*   - *If the person was accompanied by someone at the time of diagnosis*   - *Or if someone accompanied him/her to the reception of the health facility...* | | | |  |
|  | *If the participant does not in care, ask why*?   - *Explore possible causes:*   - *If there was a problem related with the circuit hospital,*   - *Or if he/she was discouraged because he/she perceived a lack of confidentiality/empathy/respect from health providers (explore stigma),*   - *Or because family responsibilities,*   - *Or because of the distance to the health facility*   - *Or any incident that occurred that prevented him/her from coming to the health facility, etc..* | | | |  |
|  | ***Ask the participant to show their HIV follow-up card***  **Did the participant show the follow-up card?** □ Yes □No | | |  |  |
|  | if 21 is No, why? □ Refusal □Lost □don’t have it □Other \|__\|__\|__\|__\|__\|__\|__\|__\|__\|__\|__\| | | | |  |
|  | **If 21 is Yes, the Hospital ID number is the same that the number on your list?**  □ Yes □ Other (Health facility different from Manhiça)  \|__\|__\| /\|__\|__\|__\|/\|__\|__\|/\|__\|__\|__\|__\|__\|  □ Outside of the Manhiça District \|__\|__\|__\|__\|__\|__\|__\|__\|__\|__\|__\|__\| | | | |  |
|  | If 21 is Yes, fill the next questions  24.1 Date of the first clinical visit \|__\|__\|-\|__\|__\|__\|-\|__\|__\| □ No date registered  □ The participant ensures that he/she make the visit, but no information was registered in the card  24.2 Date of the last clinical visit 1. \|__\|__\|-\|__\|__\|__\|-\|__\|__\| □ No date registered  □ The participant ensures that he/she make the visit, but no information was registered in the card  24.3 Date of the penultimate clinical visit 2. \|__\|__\|-\|__\|__\|__\|-\|__\|__\| □ No date registered  □ The participant ensures that he/she make the visit, but no information was registered in the card  24.4 State of preservation of the card  □ Good preserved □ Ruined □ It is not the first card. Good preserved  □ It is not the first card. Card ruined  □ Non official card □ Other \|__\|__\|__\|__\|__\|__\|__\|__\|__\| | | | |  |
|  | **Did the participant ever make the first appointment with the clinician?**  □ Yes □No □ Don’t know | | | |  |
|  | If 25 is Yes, what did he/she encouraged to go to the visit?   - *Explore the potential facilitators who helped the participant come to the consultation,*   *- If he/she have been afraid/ashamed and what helped him/her to do...*  *- If the person has already shared the result with someone in their home/environment. Who these people are, how they reacted (what they said, what they did, etc.).* | | | |  |
|  | *If the participant does not make the first appointment with the clinician, ask why*?     - *Explore possible causes:*   - *If there was a problem related with the circuit hospital,*   - *Or if he/she was discouraged because he/she perceived a lack of confidentiality/empathy/respect from health providers (explore stigma),*   - *Or because family responsibilities,*   - *Or because of the distance to the health facility*   - *Or any incident that occurred that prevented him/her from coming to the health facility, etc..* | | | |  |
|  | **Have you ever had blood drawn?** □ Yes □No □Don’t know | | | |  |
|  | *If 28 is Yes, what is the participant's perception of the control of his/her health in the laboratory?*   - *Explore the possible facilitators who helped the participant come and draw blood. Does the participant think it is important to do the lab tests?* - *Explore knowledge about the CD4s* | | | |  |
|  | *If the participant does not go to have blood drawn, ask why*?     - *Explore possible causes:*   - *If there was a problem related with the circuit hospital,*   - *Or if he/she was discouraged because he/she perceived a lack of confidentiality/empathy/respect from health providers (explore stigma),*   - *Or because family responsibilities,*   - *Or because of the distance to the health facility*   - *Or any incident that occurred that prevented him/her from coming to the health facility, etc..* | | | |  |
|  | Clinical HIV follow up and treatment | | |  |  |
|  | **Does the participant continue to come to the health facility to their clinical follow-up visits?**  □ Yes □No □Don’t know  31.1 If yes, where? □ HF Manhiça □ HF Maragra □ HF Palmeira/Nwamatibjana  □ HF Xinavane □ HF Taninga □ Xai-Xai Hospital  □ HF Munguini □ HF Calanga □ HF 3 Fevereiro  □ HF Ilha Josina □ HF Xibukutsu □ HF Malavel  □ HF Maluana □ HF Marracuene □ Maputo City  □ Other \|__\|__\|__\|__\|__\|__\|__\|__\|__\|__\|__\| | | |  |  |
|  | Se 31 Yes, what is the participant perception about adherence?   - *Explore whether the participant thinks it is important to continue coming to the hospital or not and why,*   *- if he/she think it's mandatory,*  *- if he/she want to go on and why,*  *- if he/she have a relative/friend/knowledge accompanying him/her, etc...* | | |  |  |
|  | *If the participant does not continue to come to the his/her clinical follow-up visits, ask why*?     - *Explore possible causes:*   - *If there was a problem related with the circuit hospital,*   - *Or if he/she was discouraged because he/she perceived a lack of confidentiality/empathy/respect from health providers (explore stigma),*   - *Or because family responsibilities,*   - *Or because of the distance to the health facility*   - *Or any incident that occurred that prevented him/her from coming to the health facility, etc..* | | |  |  |
|  | **If 31 is Yes, has the participant ever stopped?** □ Yes □ No □ Don’t know  34.1 If Yes, explore:   - *If there was a problem related with the circuit hospital,*    - *Or if he/she was discouraged because he/she perceived a lack of confidentiality/empathy/respect from health providers (explore stigma),*   - *Or because family responsibilities,*   - *Or because of the distance to the health facility*   - *Or any incident that occurred that prevented him/her from coming to the health facility, etc..* - *Explore what motivates him/her to re-engaged in care* | | |  |  |
|  | **Did the clinicians ever say that the participant needed to start taking HIV treatment?**  □ Yes □No □ Don’t know  35.1 If Yes, did the participant start? □ Yes □No □ Don’t know | | |  |  |
|  | **If 35.1 is Yes, is the participant currently going to the HF to take the HIV treatment?**  □ Yes □No □ don’t know | | |  |  |
|  | If 36 is Yes, what is the participant perception about adherence?   - *Explore whether the participant thinks it is important to continue with the treatment or not and why,*   *- If he/she think it's mandatory,*  *- if he/she want to continue on treatment and why,*  *- how does he/she feel now about ART, etc.?* | | |  |  |
|  | If 36 is No, and the participant stopped coming to the pharmacy, ask why?   - *Explore possible causes:*   - *If there was a problem related with the circuit hospital,*   - *Or if he/she was discouraged because he/she perceived a lack of confidentiality/empathy/respect from health providers (explore stigma),*   - *Or because family responsibilities,*   - *Or because of the distance to the health facility*   - *Or any incident that occurred that prevented him/her from coming to the health facility, etc..*   - *how the participant felt about ART (physical aspects, work, family, relationships), etc..* | | |  |  |
|  | **If 35 is Yes, has the participant ever stopped?** □ Yes □No □Don’t know  39.1 If Yes, explore:   - *If there was a problem related with the circuit hospital,*    - *Or if he/she was discouraged because he/she perceived a lack of confidentiality/empathy/respect from health providers (explore stigma),*   - *Or because family responsibilities,*   - *Or because of the distance to the health facility*   - *Or any incident that occurred that prevented him/her from coming to the health facility, etc..* - *Explore what motivates him/her to re-engaged in treatment* | | |  |  |
|  | Cost-effectiveness | | |  |  |
|  | Have you stopped doing your main occupation in the last month due to illness?  □ Yes □No □Don’t know | | | |  |
|  | If 40 is YES, how many days? \|__\|__\| days | | | |  |
|  | 42.1 Has the participant stopped doing his/her occupation due to illness since the HIV diagnosis?  □ Yes □No □Don’t know  42.2 If 42.1 is YES, how many times □ 1 tas vezes o CARE - NSENTIMENTO INFORMADO, dar numero de estudoples possiveis respostas, que o conselheiro prencha em funcao □ 2 □ 3 □ > 3 □Don’t know tas vezes o CARE - NSENTIMENTO INFORMADO, dar numero de estudoples possiveis respostas, que o conselheiro prencha em funcao tas vezes o CARE - NSENTIMENTO INFORMADO, dar numero de estudoples possiveis respostas, que o conselheiro prencha em funcao | | | |  |
|  | END | | | |  |
|  | Counselor code \|__\|__\|__\| Date \|__\|__\|- \|__\|__\|__\|-201\|__\| | | | | |
|  | Recording name \|__\|__\|__\|__\|__\|__\|__\|__\|__\|__\|__\|  ***Thank the participant for his or her participation.***  ***In the case of abandoned participants, end the positive counseling session and***  ***DELIVER THE LOST TO FOLLOW-UP REFERENCE GUIDE.*** | | | | |
