# Supplementary material for: Tipping the balance towards long-term retention in the HIV care cascade: A mixed methods study in southern Mozambique
Source: PLoS One. 2019 Sep 27;14(9):e0222028. doi: 10.1371/journal.pone.0222028 (PMC6764678; doi:10.1371/journal.pone.0222028)
Supplement: S1 Fig — (DOCX) [file pone.0222028.s003.docx]

**Supporting information**

**Methods**

**S1 Fig. Flowchart of the questionnaire and semi-structured interview performed for all eligible participants found and who consented (n=112).** The flow of questions with the open-end questions highlighted in red. For all questions first a yes/no/don’t know answer was required. Depending on that answer, the counselor explored barriers or facilitators that interfere in his/her continuation in care at that specific step of the HIV cascade.


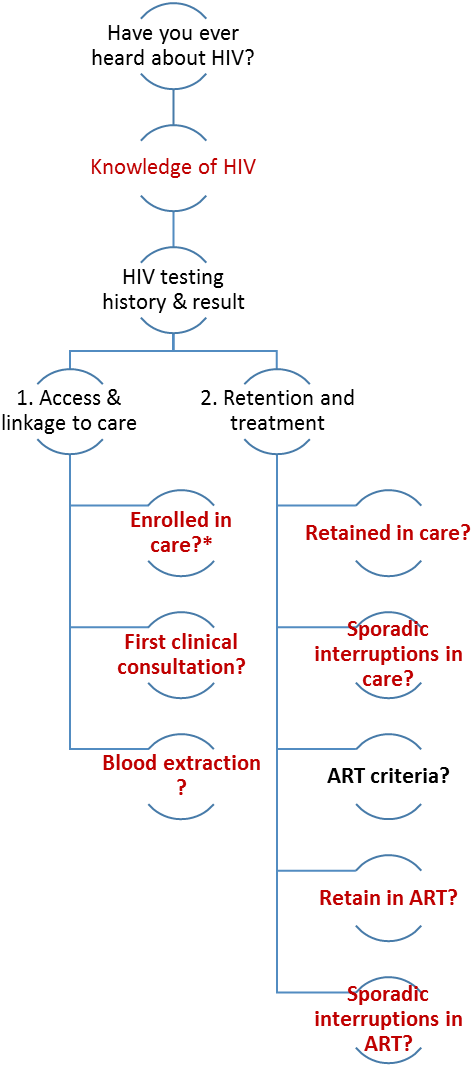


If individual reported being HIV negative or never accessing or linking to care, interview ends here

If individual reported that the clinician told them to start ART, the interview continues until the end

1. Have you ever been tested for HIV?
2. What was the result?

If individual reports being negative or never tested after HIV counseling:

1. The counsellor offers an HIV test.
